# A combined convolutional and recurrent neural network for enhanced glaucoma detection

**DOI:** 10.1038/s41598-021-81554-4

**Published:** 2021-01-21

**Authors:** Soheila Gheisari, Sahar Shariflou, Jack Phu, Paul J. Kennedy, Ashish Agar, Michael Kalloniatis, S. Mojtaba Golzan

**Affiliations:** 1 Vision Science Group, Graduate School of Health, University of Technology Sydney, Sydney, Australia; 2 Centre for Eye Health, School of Optometry and Vision Science, University of New South Wales, Sydney, Australia; 3 Center for Artificial Intelligence, Faculty of Engineering and Information Technology, University of Technology Sydney, Sydney, Australia; 4 Department of Ophthalmology, Prince of Wales Hospital, Sydney, Australia; 5 School of Optometry and Vision Science, University of New South Wales, Sydney, Australia

**Keywords:** Eye diseases, Diseases

## Abstract

Glaucoma, a leading cause of blindness, is a multifaceted disease with several patho-physiological features manifesting in single fundus images (e.g., optic nerve cupping) as well as fundus videos (e.g., vascular pulsatility index). Current convolutional neural networks (CNNs) developed to detect glaucoma are all based on spatial features embedded in an image. We developed a combined CNN and recurrent neural network (RNN) that not only extracts the spatial features in a fundus image but also the temporal features embedded in a fundus video (i.e., sequential images). A total of 1810 fundus images and 295 fundus videos were used to train a CNN and a combined CNN and Long Short-Term Memory RNN. The combined CNN/RNN model reached an average F-measure of 96.2% in separating glaucoma from healthy eyes. In contrast, the base CNN model reached an average F-measure of only 79.2%. This proof-of-concept study demonstrates that extracting spatial and temporal features from fundus videos using a combined CNN and RNN, can markedly enhance the accuracy of glaucoma detection.

## Introduction

Glaucoma is a chronic disease associated with age. It is described as an ocular disorder that encompasses a group of progressive optic neuropathies with established pathophysiological changes at the optic nerve head (ONH)^1^. The glaucoma continuum describes the optic neuropathy associated with progressive glaucomatous damage as RGC loss during the initial and asymptomatic stages of the disease followed by detectable retinal nerve fibre layer (RNFL) thinning and ultimately visual field (VF) defects^2^. Previous studies have shown that, in untreated patients, due to missed diagnosis, the risk of progressing from ocular hypertension to unilateral vision loss (i.e., legal blindness) is 1.5% to 10.5%^3^. Accordingly, early diagnosis is vital to prevent disease progression and vision loss.

Fundus photographs provide a 30-degree image of the ONH and an opportunity to quantify a number of glaucoma specific morphological features^4^, including neuroretinal rim loss, and an increased cup-to-disc ratio^5^. Despite the computer-aided approach for objective assessment of these features, there is well known inter- and intra- observer variation in analysing images obtained from the ONH^6^. Another major limitation of single fundus imaging is that it does not reveal markers associated with blood flow dysregulation, a well-established phenomenon in glaucoma^7^. To overcome this limitation, a number of recent studies have focused on analysing fundus videos (i.e. image sequences) in search of markers associated with blood flow. Through analysing fundus videos, temporal features such as alteration in the amplitude of spontaneous venous pulsations (SVP)^8^ and blood column variations^9^ can be obtained, both of which have been linked to glaucoma^10–12^. Taken together, it is imperative to incorporate both spatial and temporal features for any approach aimed at glaucoma assessment.

Computer aided diagnosis (CAD) systems play an important role in making an accurate, reliable and fast diagnosis of glaucoma^13^. Recent advances in computational power capabilities have allowed implementation of convolutional neural networks (CNN) facilitating autonomous classification of glaucoma based on complex features derived from thousands of available fundus images. The specificity and sensitivity of these models range between 85 to 95%, with the transfer-trained models (as opposed to native trained) achieving the highest performance in separating glaucoma from healthy fundus images^14–16^. While these experimental results show that pre-trained CNNs have the ability to achieve superior results in classifying glaucomatous fundus images, the complexity of glaucoma pathology poses unique challenges for CNNs trained on fundus images only. More specifically, population differences in optic disc size and colouration^17^ as well myopic discs^18^ are a potential source of errors for these networks as they have strong correlations with critical ONH structural parameters such as cup-disc ratio and neuroretinal rim thickness relevant to glaucoma diagnosis. Accordingly, such networks can only reach certain levels of accuracy and precision without compromising on false positive rates.

As glaucoma encompasses a range of spatial and temporal features, for any autonomous approach, it is essential to treat it as a video classification problem, rather than a static image problem. By taking such approach, spatial features such as changes to ONH morphology as well as temporal features such as amplitude of spontaneous venous pulsations are taken into account. In this study, we propose a novel approach through which a combined CNN and recurrent neural network (RNN) is used to extract spatial and temporal features embedded in fundus videos to detect glaucoma.

## Methods

### Data collection

Fundus images and videos were collected from 489 and 206 participants attending the Centre for Eye Health (University of New South Wales, Sydney, Australia), Marsden Eye Clinic, and Vision Research Clinic (UTS). For fundus videos, participants had a bilateral dilated funduscopy and a minimum 5-s recording (30 frames per second at a 46/60 degrees’ field of view of the retina, and 2.2 image magnification) centred on the optic disc (Table 1). All subjects were required to be > 18 years of age, visual acuity better than 6/12, and have sufficiently clear ocular media for accurate capturing of fundus photos and videoscopy.

**Table 1:** Participant demographics and data distribution.

	Glaucoma	Normal
Number of participants	379	316
Gender (female)	158	151
Gender (male)	221	165
Average age (years, SD)	65.1 ± 13	47 ± 15

We included patients with glaucoma who had primary open-angle (either high- or low-tension glaucoma), pseudoexfoliative or pigmentary glaucoma. Glaucoma patients were diagnosed clinically on the basis of characteristic structural losses observed on dilated stereoscopic examination of the optic nerve head (including but not limited to the following features: increased cup-to-disc ratio, cup-to-disc ratio asymmetry, localised or generalised neuroretinal rim thinning or notching, deepening of the optic cup, and glaucomatous disc haemorrhages) with or without concordant visual field defects on conventional white-on-white standard automated perimetry (Humphrey Field Analyzer, 24-2 SITA-Standard; Carl Zeiss Meditec, Dublin, CA). Glaucomatous visual field defects were not attributable to other diseases of the visual pathway and were defined as any of the following: three or more contiguous points of sensitivity reduction with a significance level of *p* < 0.05, of which at least one point is significant at the *p* < 0.01 level, on the pattern deviation map; a pattern standard deviation result with a significance level of *p* < 0.05; or three consecutive Glaucoma Hemifield Tests that were outside normal limits. Elevated intraocular pressure was not required for a diagnosis of glaucoma. In all cases, a glaucoma specialist was responsible for diagnosis of glaucoma.

Normal subjects were required to have a normal visual field (pattern standard deviation not less than *p* < 0.05; Glaucoma Hemifield Test within normal limits; and no cluster of points of depression as described above), normal ocular health on comprehensive dilated fundus examination and intraocular pressure below 22 mmHg. A glaucoma specialist was also involved in identifying these patients as ophthalmically normal (i.e. no glaucoma). For all subjects, optical coherence tomography was used as an adjunct to the diagnostic process, but no specific numerical cut-offs were used to determine whether or not a patient had glaucoma.

This study was performed in accordance with the guidelines of the tenets of the Declaration of Helsinki and approved by the University of New South Wales and University of Technology Sydney’s Human Research Ethics Committee. Informed consent was obtained from each subject following an explanation of the nature of the study.

### Preprocessing of data

**Figure 1:**
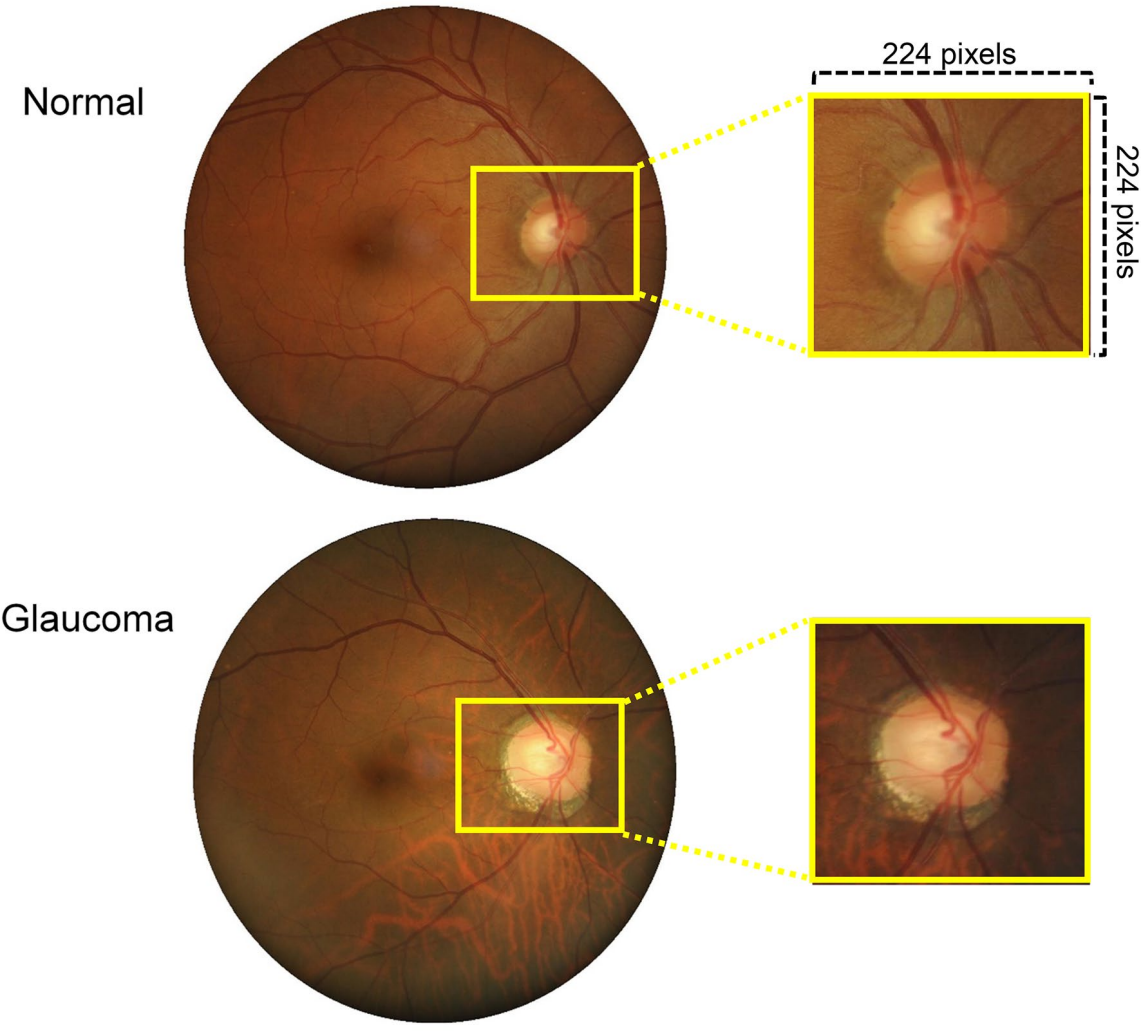
Examples of the original and cropped fundus images. All images were labeled as normal or glaucoma.

Prior to developing the deep leaning architecture, one of the authors (SG) normalized all the images and frames and then marked the center of the optic disc in all of the frames in each video. An automated algorithm (custom written in MATLAB, The Mathworks, Massachusetts, USA) was then applied to crop all images to a square region centered on the optic disc and limited to two disc diameters surrounding the manually marked disc center. This approach was chosen as glaucoma-specific static and dynamic features are mainly manifested on retinal tissue surrounding the ONH. Furthermore, a previous study by Orlando and colleagues has shown that cropping images around the optic disc is more efficient than using the whole images for training CNNs^19^. Finally, we employed a data augmentation procedure to solve the imbalance issue in our dataset and to increase the amount of normal images in the training set. The training images were augmented through random rotation, zooming and horizontal flipping. As the default size of the input images to the network is 224 × 224, we resized the images to match this specification (Fig. 1).

### Convolutional neural network (CNN): extracting spatial features from retinal images

**Figure 2:**
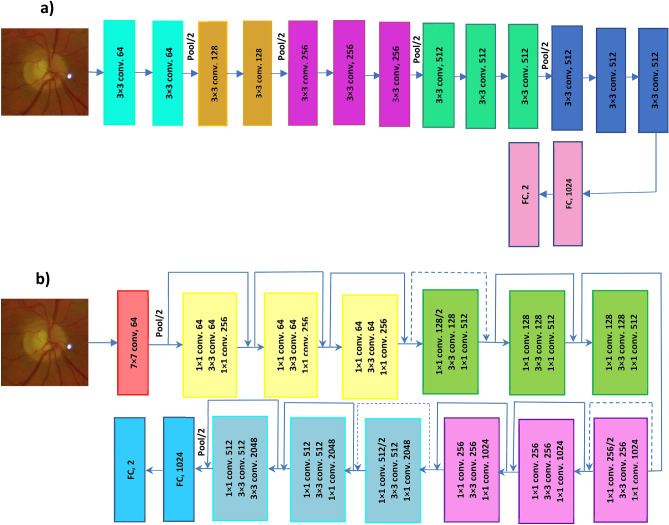
Overall framework of convolutional neural networks. (**a**) VGG16, (**b**) ResNet50.

We evaluated two different deep learning architectures in this study: VGG16^20^ and ResNet50^21^. These architectures were selected as they have been widely used for medical image classification tasks^22^. Both networks have a similar architecture and comprise of convolutional layers, pooling layers and a final fully connected layer that produces a label for the input image (Fig. 2). The depth of VGG16 is 16 layers (13 convolutional layers and 3 fully connected layer). ResNet50 contains 50 layers (16 residual layers and 2 fully connected layers, each residual layer contains 3 convolutional layers). The input of the networks was set to 224 × 224 pixel images that are passed through a stack of convolutional layers. The convolutional layers filter images with a kernel size of 3 × 3. Each convolutional block, a group of convolutional layers, is followed by a max-pooling layer. Applying the max-pooling layer not only allows the higher layer outputs to be resilient to subtle changes in the input, but also reduces the overall computational cost. A stack of convolutional layers is followed by fully connected (FC) layers to map the convolutional features to the feature vectors. The CNNs act like a feature extractor that extract static features of the input image.

### Recurrent neural network: extracting temporal features from retinal videos

Recurrent neural networks (RNN) are specifically designed to identify patterns in sequences of data or, in our scenario, images. In a traditional neural network, all inputs are treated as being independent of each other. The limitation of such approach is in tasks that require the network to remember events from previous data, such as predicating a word in a sentence or predicting a frame in a video. As RNNs perform the same task for every element of a sequence, they are called *recurrent*. In these networks, the output depends on previous computations. Moreover, these networks have a “memory” which captures information about what has been calculated so far. The most popular RNN network is Long Short Term Memory (LSTM), originally proposed by Hochreiter and Schmidhuber^23^, to learn long term dependencies. The superior performance of LSTM in language translation and speech processing has made it the first RNN of choice for tasks that feature extraction from a sequence of data is required (i.e. videos).

**Figure 3:**
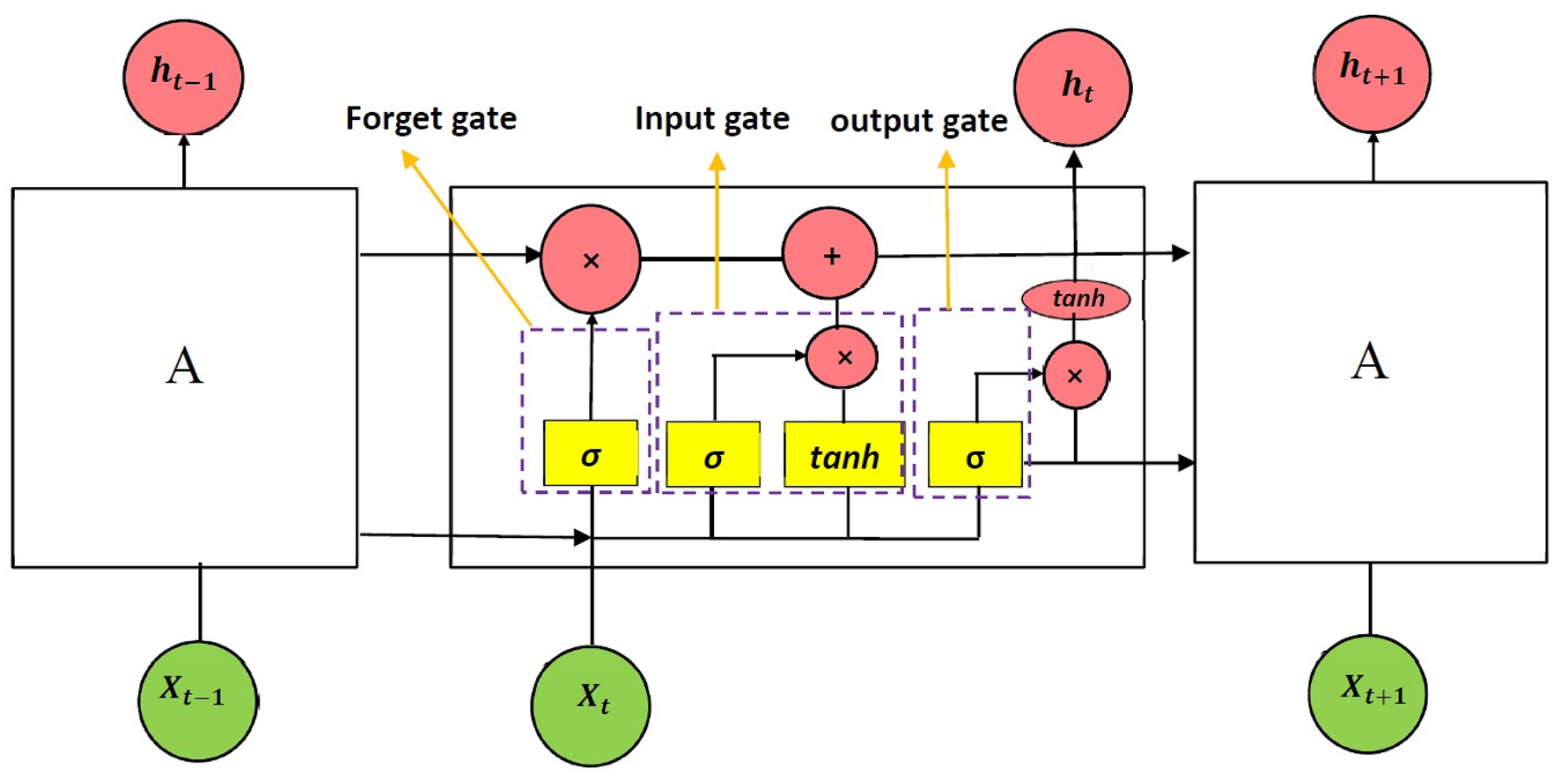
Structure of the Long Short Term Memory (LSTM), encompassing the three gates; forget, input and output. *X* denotes the input of the memory, *h* is the prediction at time t, and *σ* is a function that selects which information to retain or forget.

The LSTM architecture comprises memory blocks with each block containing a number of memory cells. Each memory cell consists of three gates: forget, input and output gate. The forget gate is an *σ* function that decides which information to retain and which to forget. The input gate decides what new information is to be stored in each cell. The output gate selects useful information from the current cell state and displays it as an output (Fig. 3).

### Combining CNN and RNN

**Figure 4:**
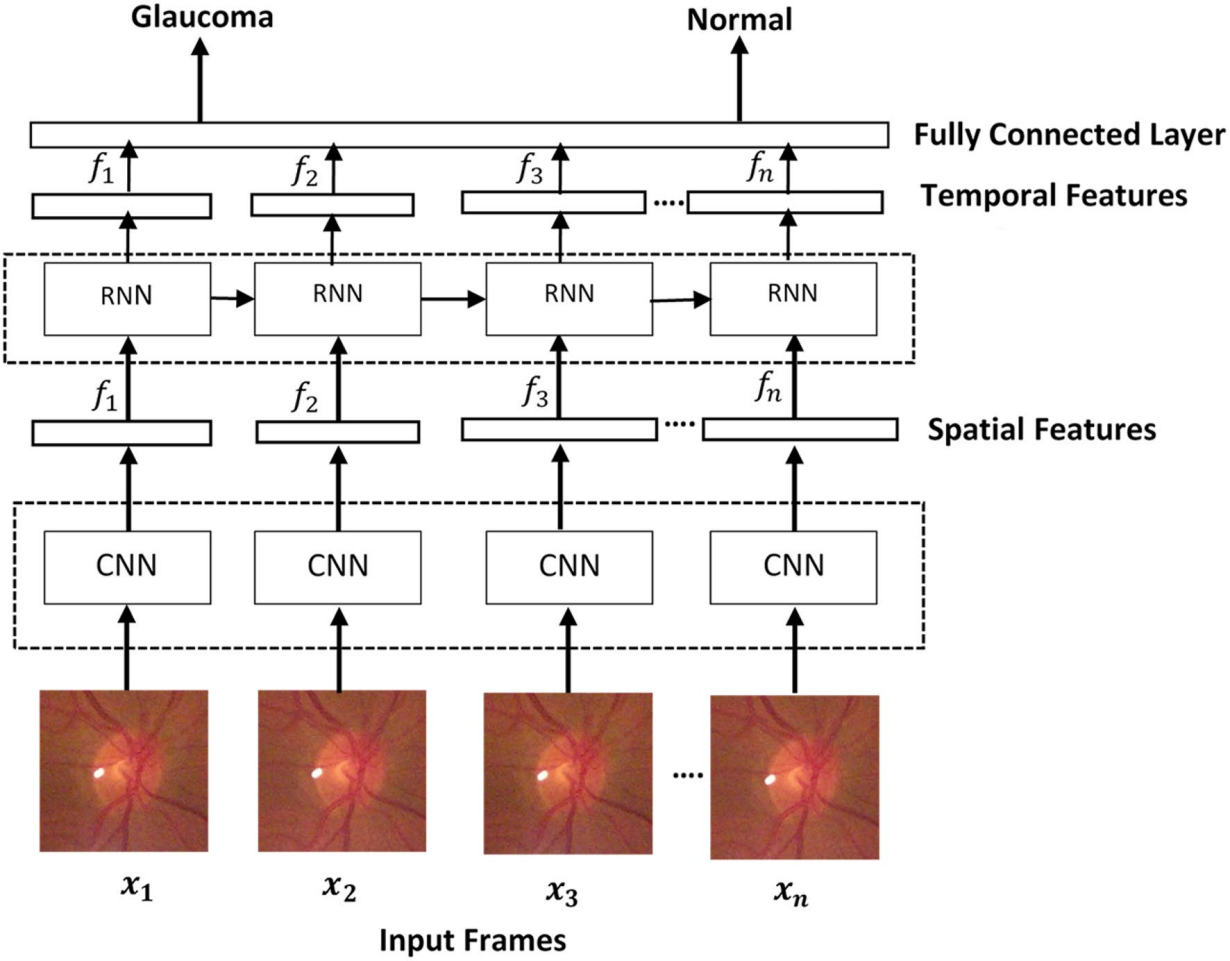
Overall framework of the fused Convolutional Neural Network-Recurrent Neural Network (CNN-RNN). The extracted spatial features from the CNN passes through the RNN to create the temporal features.

We developed a combined CNN (VGG16 and ResNet 50) and RNN (LSTM) architecture to extract spatial and temporal features, respectively. The overall framework of our approach is shown in Fig. 4. Each video is converted into sequential images and passed onto the CNN to extract spatial features. The outputs are then passed into a recurrent sequence learning model (i.e. LSTM) to identify temporal features within the image sequence. The combined features are finally passed on to a fully connected layer to predict the classification for the full input sequence.

### Network training

We evaluated the networks described earlier using transfer learning. In transfer learning, as opposed to native training, model weights are initialized based on the ImageNet dataset, a large benchmark dataset in object category classification and detection on hundreds of object categories and millions of images. Transfer learning is to initialize the model weights based on a huge general image dataset, except for the fully connected layers which are trained on our constructed fundus dataset. To train and evaluate the models, we divided the dataset randomly into three subsets: training (85% of total images), testing (10% of total images), and validation (5% of total images) and quantified the F-measure, precision and recall. This procedure was repeated ten times and the average of F-measures, precisions and recalls are reported. To ensure a homogenous training and testing, all images and videos from an individual participant were included in the same subset. In other words, all the images and videos used for testing the network were not “seen” by the network as part of the training and validation step.

### Local interpretable model-agnostic explanations: uncovering the black box

We employed the Local Interpretable Model-Agnostic Explanations (LIME) algorithm to visualize sections of the image that the model is using to produce its final prediction. Briefly, LIME works based on creating a new dataset that contains permuted samples and the associated predictions. The new dataset is then used to train a new model which is weighted by the proximity of various features in the input image to the feature of interest. As a result, the weights are continuously updated based on whether they are contributing to the prediction or are against it. The fully trained new model is then used to interpret and explain the predictions^24^.

### Network evaluation

For each of the models; VGG16, Resnet50, VGG16 + LSTM and Resnet50 + LSTM, the performance was evaluated on the training and test datasets using sensitivity, specificity, and F-measure. The area under the receiver operating characteristics curve (AUC) was also used to evaluate the performance of each model.

## Results

### The combination of VGG16 and LSTM achieved the highest performance

To determine the best performance for each model, we ran several experiments varying the number of epochs, batch size and learning rate. A batch size of eight, 20 epochs, and a learning rate of 0.001 were determined as the optimum values to achieve the best performance. Table 2 summarizes the sensitivity, specificity and F-measure achieved for each of the models, using the aforementioned parameters. Our results show that the combination of VGG16 and LSTM achieved the highest accuracy, outperforming the models trained and tested on spatial features only.

**Table 2:** Performance of the various models on the total dataset.

Models	Sensitivity	Specificity	F-measure (%)
**CNN**			
VGG16	0.59 ± 0.1	0.65 ± 0.23	70.3 ± 5
Resnet50	0.71 ± 0.19	0.76 ± 0.13	79.2 ± 11.6
**CNN + RNN**			
VGG16 + LSTM	0.95 ± 0.02	0.96 ± 0.02	96.19 ± 1.7
Resnet50 + LSTM	0.89 ± 0.04	0.97 ± 0.02	94.03 ± 2.1

As our overall database had a significant age difference between the glaucoma and normal group, a subsequent analysis was performed on a subgroup of age-matched glaucoma and normals to determine whether age may have a likely effect on the end results. A total of 23 glaucomatous fundus videos (obtained from 22 glaucoma participants, 67 ± 12 years) and 26 normal fundus videos (obtained from 17 healthy participants, 64 ± 18 years) were selected and tested using each of the models (no significant difference in age; *p* > 0.05). Table 3 summarizes the sensitivity, specificity and F-measure achieved for each model. Consistent with earlier results, aggregation of VGG16 and ResNet50 with LSTM achieved the higher F-measure, sensitivity and specificity, with the combined VGG16 and LSTM achieving the highest performance amongst all the models.

**Table 3:** Performance of the various models on the age-matched dataset.

Models	Sensitivity	Specificity	F-measure (%)
**CNN**			
VGG16	0.59	0.55	56.4
Resnet50	0.72	0.70	70.3
**CNN + RNN**			
VGG16 + LSTM	0.94	0.86	89.9
Resnet50 + LSTM	0.88	0.79	83.9

### Combined CNN and RNN models achieved significantly higher performance than CNNs alone

An inter-group analysis was performed to evaluate the performance of each model in comparison to others. A one-way ANOVA applied to the average sensitivity, specificity and F-measure revealed a significant difference amongst all models (*p* < 0.0001, *p* < 0.001, *p* < 0.0001, respectively). Post-hoc analysis showed that a combined CNN and RNN, outperformed sole CNN models across all measures of sensitivity, specificity, and F-measure. The combination of VGG16 and LSTM achieved the highest performance in comparison to VGG16 for separating glaucoma from healthy image and videos, with a sensitivity of 0.95 (vs. 0.59; *p* < 0.0001) , specificity of 0.96 (vs. 0.65; *p* < 0.0001), and F-measure of 96 (vs. 70; *p* < 0.0001). (detailed post-hoc results available in supplementary file, tables S1-S3).

### Combined VGG16 and LSTM achieved the highest AUC in separating glaucoma from healthy eyes

**Figure 5:**
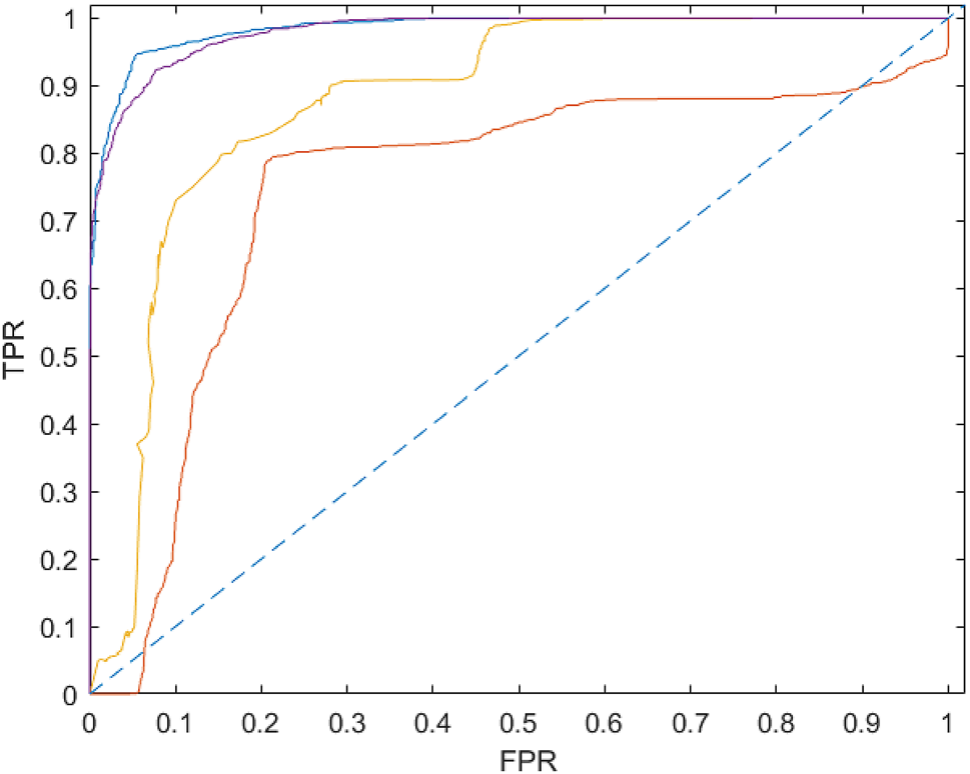
The receiver operating characteristic curves (AUC) for all the different models.

To evaluate each model’s performance in separating glaucoma from healthy eyes, we compared the area under the Receiver Operating Characteristic Curves (AUC). The VGG16 and ResNet50 models achieved an AUC of 0.6 and 0.83, respectively. However, these values increased to 0.99 and 0.97 for VGG16 + LSTM and ResNet50 + LSTM, respectively. Consistent with previous results, the combined VGG16 and LSTM achieved the highest AUC (Fig. 5).

**Figure 6:**
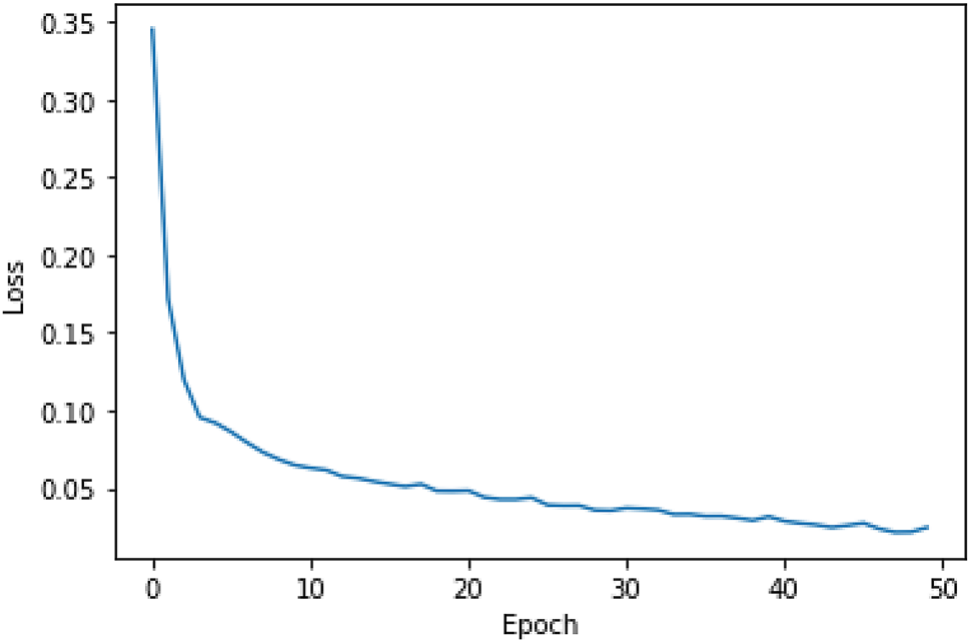
The loss over the epochs for the train dataset.

**Figure 7:**
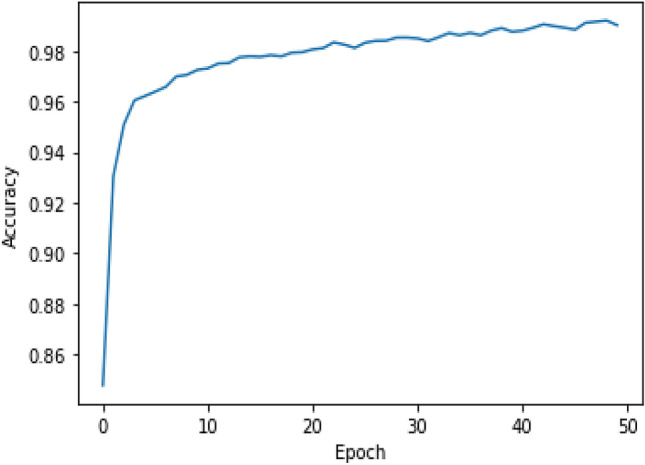
The accuracy over the epochs for the train dataset.

The loss and accuracy plots over the epochs for the train dataset are shown in Figs. 6 and 7. From the curves, we can see that our methods have a good behavior of convergence.

### Temporal features drive the accuracy of the end prediction

To evaluate whether the increased accuracy observed in the combined models are driven by temporal features (and is not due to the increased sample size as result of multiple frames), we trained the combined models using fundus images, tested with sequential frames, all whilst eliminating the sequential frames from the training set. A significant decrease in the performance of both combined models was noted, further confirming that temporal features played a significant role in achieving the higher performance shown earlier (Table 4).

**Table 4:** Performance of combined CNN with RNN trained on images and tested on sequential images.

Models	Sensitivity	Specificity	F-measure (%)
VGG16 + LSTM	0.51	0.58	63
Resnet50 + LSTM	0.76	0.64	82

### Vascularized regions were predominantly influential in predicting end-classification

**Figure 8:**
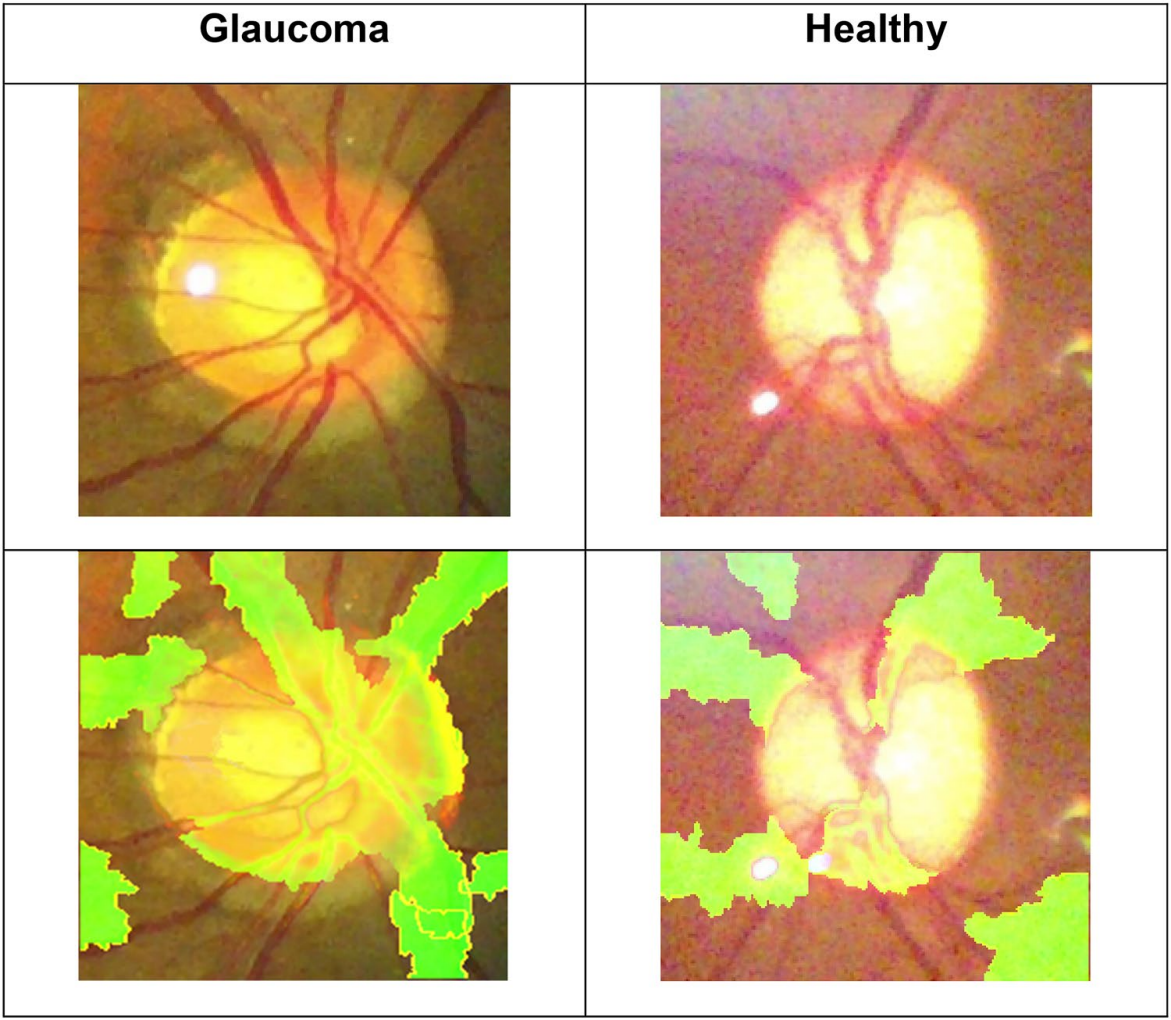
Average intensity map (60 sequential frames) from the LIME algorithm applied to the combined models showing significant regions of the image used to predict glaucoma and healthy. Regions with brighter intensity (green) indicate a larger impact compared to regions with lower intensity (yellow).

The LIME algorithm was employed to uncover regions of the image that the combined CNN/RNN model is using to extract spatial and temporal features and to produce its final class prediction (i.e., glaucoma or normal). The vascularized regions of superior and inferior retina were identified as regions of “influence” the VGG16 + LSTM has utilized in determining the final classification (Fig. 8). As we applied CNN + RNN on sequential frames of a video, the LIME algorithm is applied on one frame of a video to visualize the difference between a healthy and a glaucoma eye.

## Discussion

In this study, we show, for the first time, that training a combined CNN and RNN on retinal image sequences, can extract both spatial and temporal features, resulting in significantly improved detection of glaucoma. More specifically, the combination of a VGG16 or ResNet50 network with an LSTM architecture, reached significantly higher sensitivity and specificity rates compared with a base VGG16 or ResNet50 network. The VGG16 + LSTM combination reached the highest performance, amongst all other networks, achieving a sensitivity of 0.95, specificity of 0.96, F-measure of 96.19% and an AUC of 0.99 in separating glaucoma from healthy eyes. The concept described here provides the first solid evidence that by treating glaucoma as a video classification task, a highly accurate and autonomous AI-based approach for glaucoma detection can be developed.

While the retinal structures are static in nature, our reasoning for improved performance of the combined model is based on the dynamic changes of retinal vasculature observed at the ONH. More specifically, there is now evidence that the amplitude of spontaneous venous pulsations (SVP) are linked to glaucomatous pathology with lower SVPs associated with thinner RNFL thickness and visual field defects^25^. While the origin of SVPs is a matter of debate, there is a consensus that the trans-laminar pressure difference is the main driver behind these pulsations. The translaminar pressure is governed by the difference in IOP and the pressure of the cerebrospinal fluid surrounding the optic nerve sheath; both of which have been linked to glaucomatous pathology^26,27^. Furthermore, using a modified photoplethysmography tool (i.e., using the green channel of a colour fundus video to visualise blood column variation in the axial direction and across all regions of the retina.), Morgan et al. have shown a reduction in blood column intensity of glaucoma patients compared to normal^9^. It is these vascular changes that manifest themselves through temporal features of a fundus video and in combination with spatial features of the ONH, result in an overall improved performance of the combined model, as demonstrated in this study.

Deep CNNs have proven to be an efficient AI-based tool in identifying clinically significant features from retinal fundus images^28–31^. Deep CNNs trained on glaucomatous fundus images have achieved a varied performance ranging from 0.75 to 0.9 in sensitivity and 0.65 to 0.97 specificity^16,32,33^ and these differences may be related to sample size. A recent study by Liu et al.^34^, using a deep CNN architecture applied to 3,788 fundus photographs, showed a 87.9% in sensitivity and 96.5% in specificity in glaucomatous disc identification. A follow-up study by Christopher et al.^15^, using a similar approach on 14,822 fundus photographs, showed a network sensitivity and specificity of 0.84 and 0.83 in distinguishing glaucoma eyes from healthy eyes. There have been other attempts at applying deep learning to a combination of retinal imaging modalities, mainly fundus images and optical coherence tomography (OCT) scans, to improve network performance. Such networks have achieved an area under the ROC curve of 0.94 in separating glaucoma from normal healthy eyes^35^. While promising, the approach requires access to both modalities and double the data, limiting scaling for large screening programs, mainly in remote and rural areas where access to OCT can be scarce. Furthermore, such approach is, again, based on spatial features only, neglecting the potential impact of temporal features. In contrast, our approach only requires a single device that can capture retinal image and videos.

Collectively, while deep learning methods have been capable of classifying glaucoma based on fundus images, they are yet to be utilised in a clinical setting mainly due to a high proportion of false positive rates and low precision. To overcome this limitation, we developed a model combining a deep spatial feature extractor (i.e. VGG16 and ResNet 50) with a model that can learn to recognize and synthesize temporal features (i.e. LSTM). By taking this approach, our aim was to combine glaucoma-specific temporal features (extracted from image sequences) with spatial biomarkers (extracted from fundus images) to significantly improve classification results compared with a model trained on fundus images only.

To compare results between base and combined models, we constructed and trained a number of architectures; VGG16, ResNet50, VGG16 + LSTM, and ResNet50 + LSTM. For all networks, a transfer training approach was used. This method was chosen mainly for two reasons. First, Christopher et al.^15^, has shown that a model encompassing transfer learning achieves a statistically significantly higher performance than natively trained models. Second, our sample size is relatively small and thus native training may not have achieved the intended results we were anticipating. All networks were trained and tested on images and sequential frames extracted from the videos. Our results showed that overall a combined CNN and RNN outperforms sole CNNs.

Amongst all the models trained, the combined VGG16 and LSTM achieved the highest performance. Intra-group analysis revealed a marginal statistically significant difference between the VGG16 and ResNet50 network (*p* = 0.04), with the latter achieving a higher F-measure. This is consistent with a previous report demonstrating higher performance of ResNet50 compared with VGG16^15^. We also observed a non-significant difference between the VGG16 + LSTM and ResNet50 + LSTM (*p* = 0.19). While ResNet50 outperformed VGG16, it was the combination of VGG16 and LSTM that achieved the highest performance. This could be attributed the network structure within each model. Our trained ResNet50 had a total of 50 layers compared with 16 layers in the VGG16. Deeper networks extract more complicated features and achieve higher accuracy. The fact that the combination of VGG16 with LSTM achieved higher performance, demonstrates that the temporal features are possibly a dominant factor in predicting output classification compared to spatial features. As the output dimension of the ResNet50 is higher than VGG16, its connection with the LSTM needs more parameters. Both networks, VGG16 + LSTM and ResNet50 + LSTM are trained with the same dataset. However, Resnet50 + LSTM requires more training data since it has more free parameters. Therefore, under similar conditions, the network with more free parameters (i.e. VGG16 + LSTM) achieves higher accuracy.

To help understand and visualize the temporal features of the image sequence the combined model is utilizing to distinguish glaucoma from healthy videos, we used the LIME algorithm. Results showed that the vascularized regions of superior and inferior retina have the greatest impact on the model prediction. This is consistent with our previous studies that showed a dynamic vascular change in glaucoma eyes^10,36^. We further evaluated the significance of temporal features on the model prediction by training the combined models using fundus images and testing with sequential images. This resulted in an average 22% decrease in the F-measure of the combined models, further proof that temporal features play a significant role in increasing model performance.

### Limitations

Our study has a number of limitations. First, our sample size is relatively small and is from a racially homogenous population. This has resulted in lower network accuracy of the stand-alone VGG16 and ResNet50 compared to other research reports. However, the primary goal of our study was not to develop a highly precise model for glaucoma detection, but rather demonstrate that adding recurrent connections to stand-alone CNNs could significantly increase a network’s performance in separating glaucoma from healthy images .Nonetheless, we acknowledge that a larger and heterogeneous sample size is required to further support our findings. Second, videos obtained from glaucoma and normal participants, encompassing our final dataset, were not age-matched. However, we performed an analysis on a subgroup of age-matched glaucoma and controls, with results confirming our initial findings; combined CNN/RNN performed superior compared with CNN-only models. Third, the ground truth was based on the clinical evaluation of two different expert clinicians, with one labelling the images and the other labelling the videos. Accordingly, we cannot determine agreement. However, given that a benchmark inclusion and exclusion criteria was used for each of the two groups, we do not anticipate a significant impact on our findings. Finally, the camera used to capture retinal fundus videos was different to that of used for capturing retinal fundus images. As a result, the effect of such approach on our results cannot be determined.

In conclusion, this study presented a novel AI-based approach for glaucoma detection based on unification of spatial (static structural) and temporal (dynamic vascular) features. By considering glaucoma as a video classification task, our proposed method demonstrated promising performance in separating glaucoma from healthy compared with models solely reliant on spatial features as their input. Static features such as disc size, cup and rim size, and cup-to-disc ratio are variable across different populations^37^, and therefore, have hindered development of models with high sensitivity and specificity^38^. The method proposed here achieved a sensitivity of 0.95 and specificity of 0.96 in identifying glaucoma and thus, may provide an impetus for large-scale screening programs. However, further evaluation on a larger, heterogeneous population is required.

## Supplementary Information

Supplementary Information 1.
