# Supplementary material for: A combined convolutional and recurrent neural network for enhanced glaucoma detection
Source: Sci Rep. 2021 Jan 21;11:1945. doi: 10.1038/s41598-021-81554-4 (PMC7820237; doi:10.1038/s41598-021-81554-4)
Supplement: Supplementary file 1 — Supplementary Information 1. [file 41598_2021_81554_MOESM1_ESM.pdf]

Supplementary Information for non-parametric Post-hoc Analysis

A COMBINED CONVOLUTIONAL AND RECURRENT NEURAL NETWORK FOR ENHANCED GLAUCOMA DETECTION

Soheila Gheisari[1*], Sahar Shariflou[1], Jack Phu[2,5], Paul J. Kennedy[3], Ashish Agar[4], Michael Kalloniatis[2,5], S.Mojtaba Golzan[1]

[1]Vision Science Group, Graduate School of Health, University of Technology Sydney

[2]Centre for Eye Health, School of Optometry and Vision Science, University of New South Wales

[3]Center for Artificial Intelligence, Faculty of Engineering and Information Technology, University of Technology Sydney

[4]Department of Ophthalmology, Prince of Wales Hospital, Sydney, Australia

[5]School of Optometry and Vision Science, University of New South Wales

**Corresponding Author*

Table S1. F-measure

Model comparison	Rank Sum Difference	Adjusted p-value
VGG16 vs. Resnet50	-9	0.7
VGG16 vs. RESNET50+LSTM	-21	0.001
VGG16 vs. VGG16+LSTM	-26	<0.0001
Resnet50 vs. RESNET50+LSTM	-12	0.2
Resnet50 vs. VGG16+LSTM	-17	<0.01
RESNET50+LSTM vs. VGG16+LSTM	-5	>0.9

Table S2. Sensitivity

Model comparison	Rank Sum Difference	Adjusted p-value
VGG16 vs. Resnet50	-10	0.5
VGG16 vs. RESNET50+LSTM	-18	<0.01
VGG16 vs. VGG16+LSTM	-28	<0.0001
Resnet50 vs. RESNET50+LSTM	-8	0.1
Resnet50 vs. VGG16+LSTM	-18	<0.01
RESNET50+LSTM vs. VGG16+LSTM	-10	0.5

Table S3. Specificity

Model comparison	Rank Sum Difference	Adjusted p-value
VGG16 vs. Resnet50	-8	0.9
VGG16 vs. RESNET50+LSTM	-25.5	<0.001
VGG16 vs. VGG16+LSTM	-22.5	<0.0001
Resnet50 vs. RESNET50+LSTM	-17.5	<0.01
Resnet50 vs. VGG16+LSTM	-14.5	0.07
RESNET50+LSTM vs. VGG16+LSTM	3	>0.9
